# Single-centre retrospective experience of complications and outcomes of computed tomography-guided lung biopsy and interventions for the management of intrathoracic infections

**DOI:** 10.1186/s13104-025-07615-3

**Published:** 2025-12-16

**Authors:** Kohei Fujita, Naoki Fujimoto, Saiki Yoshimura, Shogo Toyama, Takanori Ito, Takuma Imakita, Issei Oi, Osamu Kanai, Yuki Yamamoto, Kiminobu Tanizawa

**Affiliations:** Division of Respiratory Medicine, Center for Respiratory Diseases, National Hospital Organization Kyoto Medical Center, 1-1, Fukakusa-Mukaihata, Fushimi, Kyoto, Japan

**Keywords:** CT-guided biopsy, Lung abscess, Catheter drainage, Respiratory infections, Lung biopsy

## Abstract

**Objective:**

To investigate the complications and outcomes of computed tomography (CT)-guided lung biopsy and interventions in the management of intrathoracic infections.

**Results:**

Twenty-nine patients were examined. Eleven (37.9%) patients were diagnosed with pulmonary nodules, and 18 (62.1%) underwent drainage of lung abscesses. The median age of the patients was 77 years, and 44.8% had a poor performance status. The major underlying diseases were hypertension and malignancies. The puncture site was mostly the right lower lobe (44.8%), whereas there were no cases of puncture in the left upper lobe. The nodule diagnostic success rate was 54.5% and the success rate of lung abscess drainage was 88.9%. The common organisms identified were *Aspergillus*, *S. pneumoniae* and *S. aureus* in the nodules and *S. intermedius* in the lung abscess drainage. Pigtail catheters were most commonly used for drainage (50%). Pneumothorax and hypoxaemia were the most common adverse events (13.8%), followed by fever (10.3%). *Clinical trial registration*: This retrospective study was registered in the UMIN Clinical Trials Registry (registration No. UMIN000055755). This clinical trial was registered on 6 October 2024 and is publicly available.

**Supplementary Information:**

The online version contains supplementary material available at 10.1186/s13104-025-07615-3.

## Introduction

In practice, computed tomography (CT)-guided lung biopsies are often performed to diagnose thoracic malignancies. The diagnostic rate of malignancy with CT-guided lung biopsy is considered high; however, the procedure is highly invasive owing to complications, such as pneumothorax [1–3]. Respiratory tract infections including lung abscesses can also present with nodular shadows that are difficult to differentiate from lung cancer and are often difficult to diagnose. There is a coherent report of CT-guided biopsies focusing on cases of infection; however, it has been shown to be useful in determining treatment strategies [4]. In addition to the bronchoscopic approach, CT-guided lung biopsy is considered an important diagnostic technique. In past years, CT-guided percutaneous catheter drainage of lung abscesses has been reported to be useful, with a success rate of approximately 80% [5, 6].

CT-guided interventions are commonly performed for lung malignancies, but less frequently for lung infections. There are few coherent reports on CT-guided lung biopsy and drainage interventions for diagnostic and therapeutic purposes in respiratory infections, and data on its usefulness and safety are scarce [4, 5]. Therefore, we retrospectively investigated these cases at our institution to assess the complications and outcomes of CT-guided lung biopsy and interventions.

## Main text

### Patients and methods

**Fig. 1:**
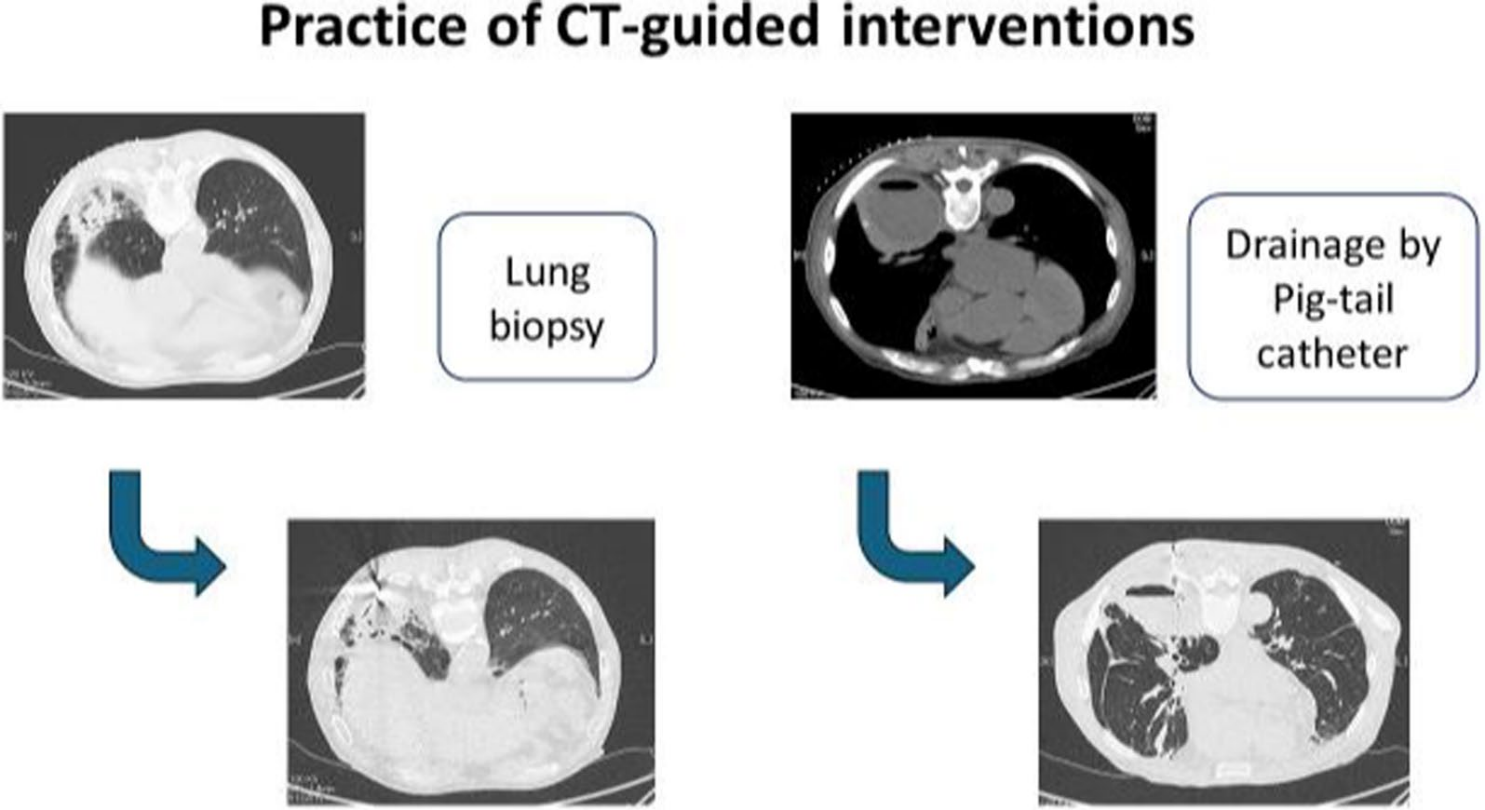
Clinical practice of CT-guided interventions

This single-centre, retrospective cohort study was conducted at the National Hospital Organization (NHO) Kyoto Medical Center (a 600-bed hospital in Kyoto, Japan). We retrospectively analysed patients who underwent CT-guided lung biopsy or lung drainage for the diagnosis or treatment of intrathoracic infections between October 2016 and August 2024. We included patients who underwent CT-guided lung biopsy or drainage for the purpose of diagnosing or treating infections at our hospital in this study. Among these patients, we excluded those who were diagnosed with conditions other than infections based on biopsy pathological results, those for whom clinical course information was not available in the electronic medical records, and those who had undergone bronchoscopy prior to the procedure. In the present study, the diagnosis of respiratory infections was based on the identification of pathogenic microorganisms. The efficacy was assessed using the pathogenic microbiological diagnostic rate of CT-guided lung biopsies, and the success rate of treatments for respiratory infections was assessed based on the improvement achieved using CT-guided drainage and antimicrobial treatment. Successful treatment was defined as an improvement in clinical symptoms, CT imaging and blood biochemistry findings (C-reactive protein and white blood cell counts normalised or improved to near-normal levels) following CT-guided drainage and removal of the thoracic drain without subsequent relapse. Improvement in CT imaging was defined as disappearance of the abscess, disappearance of the abscess cavity, or disappearance of clinical symptoms such as fever and respiratory symptoms even if there was a residual abscess cavity. CT-guided lung biopsies or drainage were performed by respiratory medicine specialists with at least 6 years of clinical experience and, as a rule, sedation was not performed during the examination. The sedation with midazolam was only performed if the patient strongly requests it before the examination. Usually, only local anaesthesia with Xylocaine was used. The CT imaging method used was intermittent imaging, and the position was checked several times during each imaging session. Clinical practice of CT-guided intervention technique was illustrated in Fig. 1. Adverse events were defined as follows: Hypoxemia was defined as a 4% drop in oxygen saturation from baseline or the need for additional oxygen delivery. Hypertension and hypotension were defined as a systolic blood pressure > 160 mmHg or a systolic blood pressure < 80 mmHg, respectively. Fever was defined as a new onset of a fever of ≥ 38 °C following CT-guided biopsy or drainage. Pain was defined as any new pain that appeared after the procedure and required treatment. In our institution, postoperative pain is treated with the use of acetaminophen on an ad hoc basis (500 mg per dose). We defined persistent pain as pain requiring the use of acetaminophen more than four times.

The severity of adverse events was assessed using the Common Terminology Criteria for Adverse Events, version 5.0. The observation period for complications was 1 month from the end of the examination. R and RStudio were used for the statistical analysis of this study. Categorical variables were analyzed using the chi-square or Fisher’s exact test, while continuous variables were first tested for normality using the Shapiro–Wilk test, with a student’s t-test if they followed a normal distribution and a Wilcoxon rank sum test if they did not follow a normal distribution (Supplemental Table S1). The NHO Kyoto Medical Center Review Board approved the study protocol (Approval No.: 24-025). This retrospective study was registered in the UMIN Clinical Trials Registry (registration No. UMIN000055755).

## Results

During the study period, there were 25 patients who underwent CT-guided lung biopsy for the diagnosis of infection and 20 patients who underwent CT-guided drainage for the treatment of infection. Of these, 14 and 2 patients underwent prior bronchoscopy, respectively. We finally reviewed the data of 11 patients who underwent CT-guided lung biopsy and 18 patients who underwent CT-guided drainage for the diagnosis and treatment of infectious diseases. A total of 29 patients were included in this analysis. The characteristics of the study participants and the statuses of their corresponding CT-guided procedures are presented in Table 1. The median age of the eligible participants was 77 years; they were predominantly male, and 44.8% had a poor performance status. The most common comorbidities were hypertension (48.3%), malignancies (41.4%), and cardiovascular diseases (37.9%). The right lower lobe (44.8%) was the most common lung puncture site, followed by the right middle (20.7%) and left lower lobes (17.2%).

**Table 1:** Characteristics of the study participants

Patients characteristics	n = 29
Age, median	77 (48–92)
Sex (male)	17 (58.6)
BMI, mean	19.7 ± 3.4
Poor PS (≥ 2)	13 (44.8)
Smoking (never)	12 (41.4)
Comorbidity	
COPD	5 (17.2)
Asthma	1 (3.4)
Cardiovascular	11 (37.9)
Hypertension	14 (48.3)
Diabetes mellitus	6 (20.7)
CKD/Haemodialysis	1 (3.4)
Cerebrovascular	4 (13.8)
Malignancy	12 (41.4)
Anticoagulant agent	3 (10.3)
Antiplatelet agent	4 (13.8)
Target lesion	
Rt upper lobe	3 (10.3)
Rt middle lobe	6 (20.7)
Rt lower lobe	13 (44.8)
Lt upper lobe	0
Lingular	1 (3.4)
Lt lower lobe	5 (17.2)
Chest wall	1 (3.4)
Type of intervention	
CT-guided lung biopsy	11 (37.9)
CT-guided drainage of lung abscesses	18 (62.1)

Data are shown number (%), mean  ±  standard deviation or median (range). *BMI* Body mass index; *PS* Performance status; *COPD* Chronic obstructive pulmonary disease; *CKD* Chronic kidney disease; *CT* Computed tomography

Table 2 lists the details of each procedure. Of the 11 patients who underwent CT-guided lung biopsy, the pathogenic microorganisms of origin were correctly identified in 6 (54.5%). The mean distance between the surface epidermis and target lesion was 34.2 ± 17.4 mm. Mean time of procedure was 19.5 ± 6.2 min. In contrast, the mean time for CT-guided drainage of lung abscess was 26.6 mm, which was significantly longer than for CT-guided lung biopsy. Drainage treatment was successful in 16 (88.9%) of the 18 patients who underwent CT-guided drainage of lung abscesses. The drainage tube was an 8-Fr pigtail catheter in nine (50.0%) patients, a 16-G intravenous cannula (Happy-Cas) in six (33.3%) patients, a 16-Fr trocar aspiration kit in one (5.6%) patient and a 20-Fr trocar catheter in two (11.1%) patients. The median duration of drain insertion was 5.5 days. The median duration of hospitalisation was 2.0 days and 21.5 days for patients who underwent CT-guided lung biopsy and CT-guided drainage, respectively. Two (18.2%) patients undergoing CT-guided lung biopsy and 15 (83.3%) patients undergoing CT-guided drainage received antimicrobials prior to testing.

**Table 2:** Detailed procedure of computed tomography-guided lung biopsy and drainage

	CT-guided lung biopsy	CT-guided drainage of lung abscesses	*P* value
	n = 11	n = 18	
Distance from the surface epidermis to target lesion, mm	34.2 ± 17.4	35.8 ± 19.9	0.9821
Median time of procedure, min	19.5 ± 6.2	26.6 ± 9.1	0.0363
Diagnosis accuracy of CT-guided lung biopsy (n = 11)	6/11 (54.5)*	–	0.0712
Treatment success of CT-guided drainage of lung abscesses (n = 18)	–	16/18 (88.9)**	
Antimicrobials use before procedure	2 (18.2)	15 (83.3)	0.00123
Type of drainage tube (n = 18)			
8-Fr pig tail catheter	–	9 (50.0)	
16-G intravenous cannula (Happy-Cas)	–	6 (33.3)	
16-Fr trocar aspiration kit	–	1 (5.6)	
20-Fr trocar catheter	–	2 (11.1)	
Median duration of drain removal, days	–	5.5 (1.0–25.0)	
Median duration of hospital stay, days	2.0 (2.0–11.0)	21.5 (4.0–85.0)	< 0.001

Data are shown number (%), mean  ±  standard deviation or median (range). *CT* Computed tomography 95% confidence interval = *25.1–84.0%, **74.0–100%

**Fig. 2:**
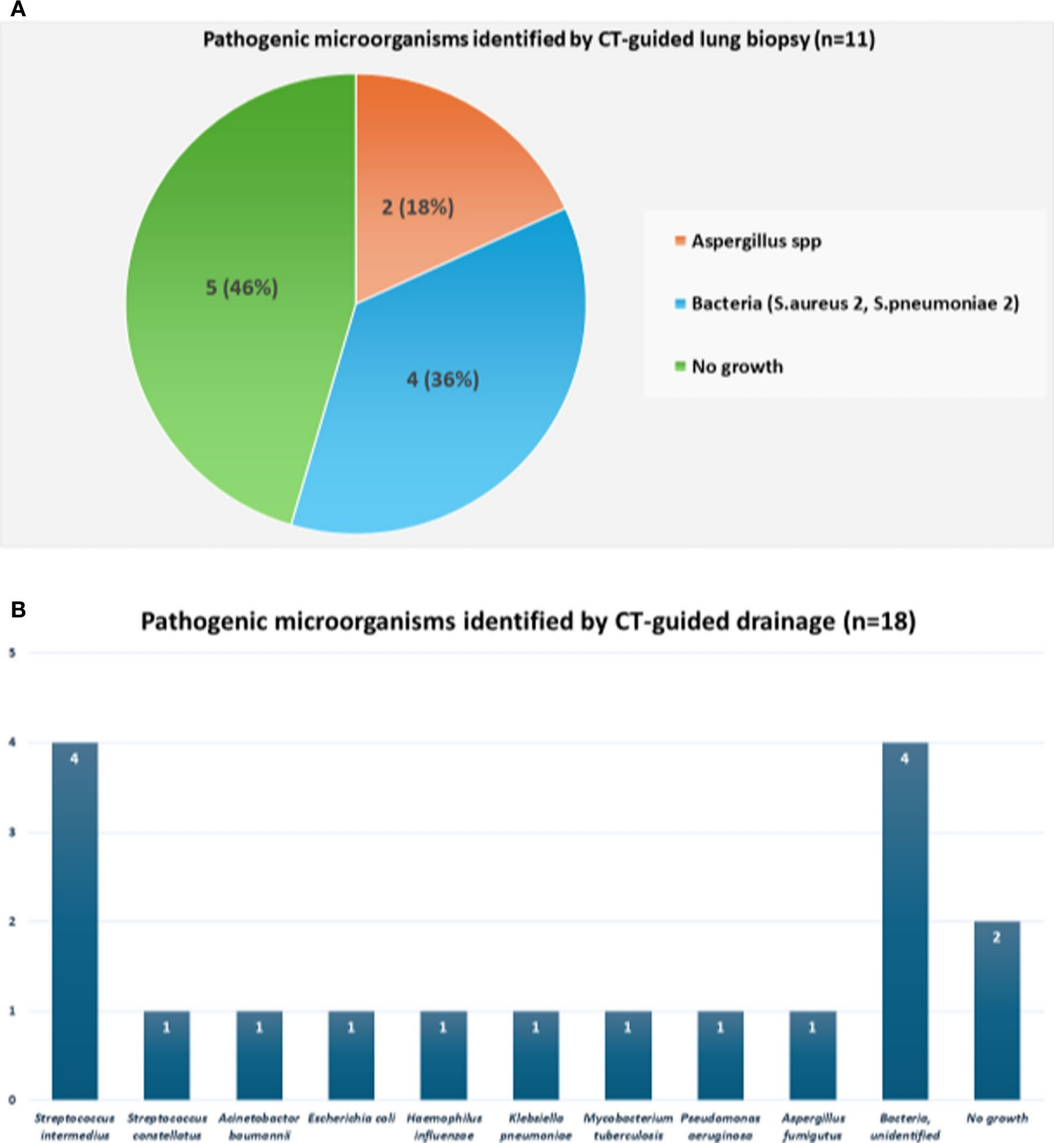
**a** A pie chart of the types of pathogenic microorganisms identified by computed tomography (CT)-guided lung biopsy. **b** a bar chart of the types of pathogenic microorganisms identified using CT-guided drainage

Figure 2a, b show the types of pathogenic microorganisms identified in the cases of CT-guided lung biopsy and drainage of lung abscesses. Figure 2a is shown as a pie chart and Fig. 2b as a bar chart. For CT-guided lung biopsy, the causative microorganisms were cultured in 6 (54.5%) of the 11 patients. *Aspergillus* was detected in two (18.2%) patients. Bacteria were detected in four (36.4%) patients, including *Streptococcus pneumoniae* in two (18.2%) and *Staphylococcus aureus* in two (18.2%). For CT-guided drainage of lung abscesses, the causative microorganisms were cultured from 16 (88.9%) of the 18 patients. *Streptococcus intermedius* was the most commonly isolated bacterium in all four (22.2%) patients. *M. tuberculosis* and *Aspergillus spp* were isolated from one (5.6%) patient each.

Table 3 shows the adverse events observed during the procedures. Pneumothorax and hypoxaemia were the most common complications (13.8% each). Only one (25%) of the four patients with pneumothorax required thoracic drainage. One patient died within 1 month of the procedure; however, this was not directly related to the procedure.

**Table 3:** Adverse events during computed tomography-guided lung biopsy and drainage

	n = 29
Complication	
Pneumothorax	4 (13.8)
Requiring drainage for pneumothorax	1 (3.4)
Duration of drainage, days	4.0
Operation	0
Haemoptysis	1 (3.4)
Hypoxaemia (4% reduction from baseline in SpO_2_)	4 (13.8)
Arrhythmia	0
Fever (2 °C increase from baseline)	3 (10.3)
Intubation	0
Infection/pneumonia	1 (3.4)
Persistent pain	1 (3.4)
Unexpected delay in discharge	0
Death within 1 month	1 (3.4)

Data are shown as number (%). SpO_2_, oxygen saturation

## Discussion

In this study, we assessed the complications and outcomes of CT-guided lung biopsy and drainage for respiratory infections. Pulmonary lesions with suspected infection are often not diagnosed by sputum examination, and bronchoscopy is often performed. However, nodular lesions and lung abscesses are often difficult to diagnose by bronchoscopy [7, 8]. A CT-guided biopsy approach may be used for these lesions, and several reports have demonstrated its usefulness [1–4, 9]. In our study, we found that the diagnostic rate of infection in CT-guided biopsies of nodular lesions was low. Previously, there have been several reports on cases in which CT-guided lung biopsy was performed for infections and a diagnosis was made, all with a diagnostic rate of approximately 50–60% and many positive reports on efficacy [3, 4]. The diagnostic rate in our study was as low as previously reported. In our institution, some pathogenic microorganisms were detected in approximately 50% of the cases, including those in which bacteria were detected, although the organisms were not identified. It is likely that cultures could not be performed for many such cases because of the high number of dead bacteria, and time may have elapsed since the formation of the abscess. Pulmonary nodules often have been formed for a long time and are often internally necrotic, whereas lung abscesses are usually fresh pus and may have a higher success rate in culturing bacteria. Laboratory interventions are required as soon as possible to identify the causative organism. This may explain why the diagnosis rate of malignancy is approximately 90%, whereas the diagnosis rate of infectious diseases is as low as 50–60%.

The results for the drainage of lung abscesses were similar to those previously reported. The success rates of lung abscess treatment with percutaneous catheters alone, without surgery, have been reported to be 83% and 84%, respectively [5, 6]. Pigtail catheters were used in half (50%) of patients in the present study. Pigtail catheters are frequently used in our institution. This is because, unlike pyothorax, lung abscesses have limited space for drain placement, and pigtail catheters are more efficient in terms of size and location. Pigtail catheters are flexible, stable and minimally invasive. Recent reports have indicated that pigtail catheters can be safely used in older bedridden patients [10]. In contrast, a single puncture with an intravenous cannula (Happy-Cas) was often performed when no indwelling was required. A single puncture may also be a good indication for patients who do not receive instructions because of dementia or delirium.

Regarding antimicrobial treatment prior to procedures, it is common practice to initiate empirical antibiotic therapy at the time of imaging diagnosis in cases of lung abscesses and pyothorax. Consequently, a significantly higher proportion of these patients receive antibiotics before further diagnostic examinations. Concerning hospitalization duration, CT-guided lung biopsies are typically required a short hospital stay, often allowing discharge after an overnight observation. In contrast, CT-guided drainage procedures necessitate the placement of a catheter, which may remain in hospital for several days to ensure adequate drainage, leading to longer hospital stays.

These differences in pre-procedural antibiotic administration and hospitalization length are inherently linked to the distinct nature and requirements of each condition and procedure. Therefore, such variations should be interpreted within the appropriate clinical context and not be overemphasized.

Complications are a concern in CT-guided biopsies, with pneumothorax and haemoptysis being the most notable [11–14]. In particular, many studies have reported a significantly higher incidence of pneumothorax in CT-guided biopsy than in bronchoscopy [12, 13, 15]. The incidence of pneumothorax is estimated to range from 16 to 27% [12, 13, 15]. Pneumothorax was also observed in 13.8% of the patients in our study, which is close to the lower limit of the previously reported incidence. In contrast, the incidence of haemoptysis in our study was low (3.4%). One reason for the low complication rate of blood sputum may be that the previous study mainly tested for malignancy, whereas in the present study, we tested for infection, which may be partly due to the lack of internal blood flow. No other serious adverse events were observed, and CT-guided lung biopsy and drainage were considered relatively easy to manage. It has been shown that there is no difference in diagnostic and adverse event rates between radiologists and chest physicians who perform CT-guided lung biopsies and drainage. Therefore, hospitals without radiologists should not hesitate to have a chest physician perform this procedure [15]. Generally, the rate of CT-guided lung biopsies for infections is low compared to that for malignancies. This may be largely due to two main factors. One is the availability of other molecular epidemiological testing methods for infectious diseases. Metagenomic techniques have made significant progress in the diagnosis of infectious diseases. Although it is now possible to identify the organisms that were difficult to identify by conventional culture, it is still difficult to determine their drug susceptibility and whether they are the true organisms of origin in mixed infections. The other is the possibility of empiric treatment even in the absence of a diagnosis. Unlike malignancies, this is an area where it is permissible to try antimicrobials without a confirmed diagnosis. However, the identification of the organisms causing the infection is very important for the so-called ‘antimicrobial stewardship’, which means treatment with the minimum number of narrow-range antimicrobials required for the minimum number of days. We believe that our current study can contribute as a diagnostic tool for difficult-to-diagnose pulmonary infections. We also believe that CT-guided lung biopsy and drainage are one of the useful procedures for the diagnosis and treatment of intrathoracic infections.

## Limitations

This study was a report of a small number of cases from a single centre and the results cannot be generalised. Retrospective studies are inherently prone to selection bias and confounding variables. Our current study has no comparator group, such as patients managed without intervention or those undergoing bronchoscopy. This makes it difficult to assess whether CT-guided approaches offer any added benefit over standard care. The small sample size can reduce the power of the study to detect meaningful differences or associations, even if trends exist. In facilities where CT imaging is not readily available, techniques such as those used in this study may be difficult to perform. The decision to perform a CT-guided lung biopsy or drainage is left to the attending physician, and there are no standardised criteria. The timing is also at the discretion of the attending physician. Furthermore, the choice of drain is at the discretion of the attending physician. The absence of standardised criteria could constitute a significant bias. This is also an area for future consideration. In our study, 18.2% of CT-guided lung biopsy patients and 83.3% of CT-guided drainage patients received antimicrobials prior to testing. This may have affected detection rates. However, CT-guided drainage patients who received more antimicrobials in advance tended to have a higher detection rate of the causative organism, which may not have an impact on lung abscesses refractory to antimicrobial treatment. Another major limitation of this study is the lack of comparators such as bronchoscopy or observational studies without intervention.

## Supplementary Information

Additional file 1.

## Data Availability

The data that support the findings of this study are available from the NHO Kyoto Medical Center Ethical Committee, but restrictions apply to the availability of these data, which were used under license for the current study, and so are not publicly available. Data are however available from the authors upon reasonable request and with permission of the NHO Kyoto Medical Center Ethical Committee.

## Abbreviations

CT: Computed tomography
NHO: National Hospital Organization

## References

[1] Takeshita J, Masago K, Kato R, Hata A, Kaji R, Fujita S, et al. CT-guided fine-needle aspiration and core needle biopsies of pulmonary lesions: a single-center experience with 750 biopsies in Japan. AJR Am J Roentgenol. 2015;204:29–34. 25539234 10.2214/AJR.14.13151

[2] Görgülü FF, Öksüzler FY, Arslan SA, Arslan M, Özsoy İE, Görgülü O. Computed tomography-guided transthoracic biopsy: factors influencing diagnostic and complication rates. J Int Med Res. 2017;45:808–15. 28415930 10.1177/0300060517698064 PMC5536670

[3] Kiranantawat N, McDermott S, Fintelmann FJ, Montesi SB, Price MC, Digumarthy SR, et al. Clinical role, safety and diagnostic accuracy of percutaneous transthoracic needle biopsy in the evaluation of pulmonary consolidation. Respir Res. 2019;20:23. 30704502 10.1186/s12931-019-0982-5 PMC6357395

[4] Haas BM, Clayton JD, Elicker BM, Ordovas KG, Naeger DM. CT-guided percutaneous lung biopsies in patients with suspicion for infection may yield clinically useful information. AJR Am J Roentgenol. 2017;208:459–63. 27845850 10.2214/AJR.16.16255

[5] vanSonnenberg E, D’Agostino HB, Casola G, Wittich GR, Varney RR, Harker C. Lung abscess: CT-guided drainage. Radiology. 1991;178:347–51. 1987590 10.1148/radiology.178.2.1987590

[6] Kelogrigoris M, Tsagouli P, Stathopoulos K, Tsagaridou I, Thanos L. Ct-guided percutaneous drainage of lung abscesses: review of 40 cases. J Belg Soc Radiol. 2011;94:191. 10.5334/jbr-btr.583 21980735

[7] Wu CC, Maher MM, Shepard J-AO. CT-guided percutaneous needle biopsy of the chest: preprocedural evaluation and technique. AJR Am J Roentgenol. 2011;196:W511–4. 21512038 10.2214/AJR.10.4657

[8] Hur J, Lee H-J, Byun MK, Nam JE, Moon JW, Kim HS, et al. Computed tomographic fluoroscopy-guided needle aspiration biopsy as a second biopsy technique after indeterminate transbronchial biopsy results for pulmonary lesions. J Comput Assist Tomogr. 2010;34:290–5. 20351523 10.1097/RCT.0b013e3181bc93ef

[9] Ezenagu OC, Gabriel GE, Saha SP. Computed tomography (CT)-guided needle biopsy of lung lesions: a single center experience. Healthcare. 2024;12:1260. 38998796 10.3390/healthcare12131260 PMC11240914

[10] Fujita K, Hirai M, Saito Z, Ito T, Imakita T, Oi I, et al. A 92-year-old patient with thoracic empyema successfully treated by <scp>CT</scp>-guided insertion of a pigtail catheter. Respirol Case Rep. 2023;11(6):e01162. 37200955 10.1002/rcr2.1162 PMC10186195

[11] Kim J, Chee CG, Cho J, Kim Y, Yoon MA. Diagnostic accuracy and complication rate of image-guided percutaneous transthoracic needle lung biopsy for subsolid pulmonary nodules: a systematic review and meta-analysis. Br J Radiol. 2021;94(1127):20210065. 34662206 10.1259/bjr.20210065 PMC8553211

[12] Kano H, Kubo T, Ninomiya K, Ichihara E, Ohashi K, Rai K, et al. Comparison of bronchoscopy and computed tomography-guided needle biopsy for re-biopsy in non-small cell lung cancer patients. Respir Investig. 2021;59:240–6. 33436353 10.1016/j.resinv.2020.12.001

[13] Tajima M, Togo S, Ko R, Koinuma Y, Sumiyoshi I, Torasawa M, et al. CT guided needle biopsy of peripheral lesions–lesion characteristics that may increase the diagnostic yield and reduce the complication rate. J Clin Med. 2021;10:2031. 34065147 10.3390/jcm10092031 PMC8126034

[14] Wu CC, Maher MM, Shepard J-AO. Complications of CT-guided percutaneous needle biopsy of the chest: prevention and management. AJR Am J Roentgenol. 2011;196:W678–82. 21606253 10.2214/AJR.10.4659

[15] Ahn JH, Jang JG. Initial experience in CT-guided percutaneous transthoracic needle biopsy of lung lesions performed by a pulmonologist. J Clin Med. 2019;8:821. 31181794 10.3390/jcm8060821 PMC6616495

