# Supplementary material for: Single-centre retrospective experience of complications and outcomes of computed tomography-guided lung biopsy and interventions for the management of intrathoracic infections
Source: BMC Res Notes. 2025 Dec 16;19:34. doi: 10.1186/s13104-025-07615-3 (PMC12821236; doi:10.1186/s13104-025-07615-3)
Supplement: Supplementary file 1 — Additional file 1. [file 13104_2025_7615_MOESM1_ESM.docx]

Supplemental Table 1.

| Categorical variables | Continuous variables | |
| --- | --- | --- |
| Chi-square or Fisher's exact test | Student's t-test | with normal distribution |
|  | Wilcoxon rank sum test | without normal distribution |

Categorical variables were analyzed using the chi-square or Fisher’s exact test, while continuous variables were first tested for normality using the Shapiro–Wilk test, with a student’s t-test if they followed a normal distribution and a Wilcoxon rank sum test if they did not follow a normal distribution.
